# Burnout Among Nursing Home Care Aides and the Effects on Resident Outcomes

**DOI:** 10.1177/10775587231220072

**Published:** 2023-12-30

**Authors:** Andrea Gruneir, Stephanie A. Chamberlain, Charlotte Jensen, Greta Cummings, Matthias Hoben, Sheila Boamah, Clarisse Bosco, Sadaf Ekhlas, Sascha R. Bolt, Tim Rappon, Whitney B. Berta, Janet Squires, Carole A. Estabrooks

**Affiliations:** 1 University of Alberta, Edmonton, Canada; 2 York University, Toronto, Ontario, Canada; 3 McMaster University, Hamilton, Ontario, Canada; 4 University of Calgary, Alberta, Canada; 5 Tilburg University, The Netherlands; 6 University of Toronto, Ontario, Canada; 7 Ottawa Health Research Institute, Ontario, Canada; 8 University of Ottawa, Ontario, Canada

**Keywords:** long-term care, aged care, professional burnout, Maslach Burnout Inventory

## Abstract

While burnout among health care workers has been well studied, little is known about the extent to which burnout among health care workers impacts the outcomes of their care recipients. To test this, we used a multi-year (2014–2020) survey of care aides working in approximately 90 nursing homes (NHs); the survey focused on work–life measures, including the Maslach Burnout Inventory (MBI) and work-unit identifier. Resident Assessment Instrument Minimum Data Set (RAI-MDS 2.0) data were obtained on all residents in the sampled NHs during this time and included a unit identifier for each resident. We used multi-level models to test associations between the MBI emotional exhaustion and cynicism sub-scales reported by care aides and the resident outcomes of antipsychotics without indication, depressive symptoms, and responsive behaviors among residents on units. In 2019/2020, our sample included 3,547 care aides and 10,117 residents in 282 units. The mean frequency of emotional exhaustion and cynicism across units was 43% and 50%, respectively. While residents frequently experienced antipsychotics without indication 1,852 (18.3%), depressive symptoms 2,089 (20.7%), and responsive behaviors 3,891 (38.5%), none were found to be associated with either emotional exhaustion or cynicism among care aides.

## Introduction

Burnout among health care workers has been of substantial concern for decades and the ongoing COVID-19 pandemic has only increased attention to burnout in both academic and non-academic media. Burnout is a physiological response to prolonged work stress and can be characterized as mental and emotional exhaustion, a distance or detachment from work, and a lack of professional competence or achievement (Leiter & Maslach, 2016). Among health care workers, burnout is associated with lower levels of job satisfaction, greater intentions to leave, elevated turnover, and poorer health and well-being (Cruz et al., 2020; Khamisa et al., 2015; Macken & Hyrkas, 2014). Health care workers with higher rates of burnout report lower perceptions of care quality and patient safety, and a decreased sense of safety culture (Halbesleben et al., 2008; Liu et al., 2018), as well as a higher likelihood of leaving care tasks undone (White et al., 2019).

Research into predictors of burnout in health care settings has shown that various elements of the work environment, including staffing levels, staff skill mix, and workload, increase the likelihood of burnout among care providers (Aiken et al., 2009, 2017; Cummings et al., 2016; Liu et al., 2018).

Despite the extensive research on burnout and the myriad ways it has been shown to impact care staff and their perceptions of care, surprisingly little research has explored the direct effects of staff burnout on patient outcomes. The majority of research either measures staff reports of care outcomes or considers burnout as part of a larger conceptual model but does not test for the direct association between burnout and patient outcomes.

In our systematic review of the literature (see Appendix Figure 1), we found one study that looked at the direct association between care staff burnout and patient or resident satisfaction with care (Leiter et al., 1998), one study on patient perceptions of care (M. Chao et al., 2016), and two studies that looked at clinical outcomes (catheter-associated and surgical site infections, and well-being including depressive symptoms) (S.-F. Chao, 2019; Cimiotti et al., 2012).

Nursing and care aide staff working in nursing homes (NHs) report a higher frequency and severity of burnout than similar staff in other settings (Kandelman et al., 2018). The pressures on NH staff have increased over time due to a more complex resident case-mix, at least in part due to a greater emphasis on aging-in-place strategies (Hoben et al., 2019), but few increases in resources or direct care staffing have accompanied the changes. Burnout among NH staff has been linked to less empathy toward people with dementia (who make up the majority of NH residents; Àstrom et al., 1990), a higher likelihood of staff exhibiting or witnessing other staff engage in abusive or neglectful behavior toward residents (Neuberg et al., 2017), and more frequent missed or rushed care tasks (Knopp-Sihota et al., 2015; Song et al., 2020). Yet, of the studies that we found that looked at associations between staff burnout and patient outcomes, only the study by Chao and colleagues (S.-F. Chao, 2019) was set in NHs where they found that higher depersonalization among nursing staff was associated with reduced resident well-being, including depressive symptoms. Our review found no studies that focused on the association between care aide burnout, specifically, and resident outcomes in NH settings. This is a notable omission because care aides provide the majority of hands-on, day-to-day care in NHs and are largely unregulated, often work more than one part-time job, frequently exposed to responsive behaviors (such as physical aggression), and typically excluded from care decision-making (Chamberlain et al., 2019; Goodridge et al., 1996). Care aides are at the frontline of resident care. Given the key role of care aides in NHs, it is important to understand how their work experience impacts residents and their outcomes. The purpose of this study was to test the associations between burnout among care aides and practice-sensitive outcomes among residents in NHs in Western Canada.

## New Contribution

In this study, we used data from a Canadian multi-provincial survey of care aides that is linked to resident-level clinical assessment data. Both the care aide survey and resident data included a care unit identifier such that we could link the unit-aggregated burnout scores among care aides to the outcomes of residents who lived in that unit. Few other studies have been able to co-locate non-physician care workers and care recipients in this way.

## Conceptual Framework

**Figure 1.**
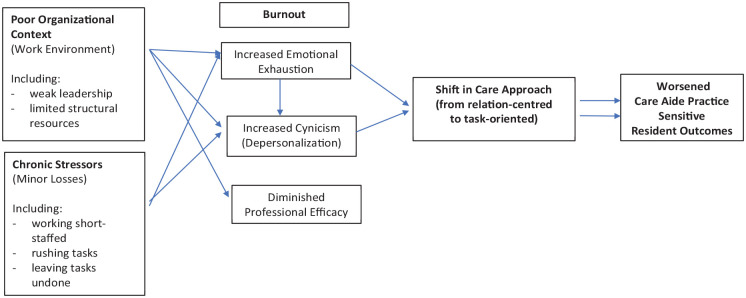
Conceptual Framework Incorporating Elements of the Nursing Worklife Model^a^ *and the Conservation of Resources Model*^b.^ Adapted from: ^a^Leiter & Laschinger (2006). ^b^Halbesleben et al. (2008).

To guide this study, we developed a conceptual framework that combined elements of the Nursing Worklife Model and the Conservation of Resources Model (see Figure 1). The Nursing Worklife Model has been used to describe the impact of the nursing work environment, usually in hospitals, on burnout among nurses. The Nursing Worklife Model consists of five factors: leadership, nurse-physician collaboration, policy involvement, staffing adequacy, and model of care. Strong leadership and nurse-centered models of care, accompanied by sufficient staffing, have been shown to have a direct association with lower burnout among hospital-based nurses (Leiter & Laschinger, 2006).

Halbesleben and colleagues (2008) suggested that the Conservation of Resources model can complement the Nursing Worklife Model by adding clarity on the inner processes of burnout (i.e., how burnout develops within an individual; Halbesleben et al., 2008). The Conservation of Resources model posits that people have a finite set of emotional resources. When those resources are strained or lost, through significant workplace events, minor chronic stressors, or insufficient return on resource investment, employees experience increased psychological stress (Hobfoll, 2001). This, in turn, results in burnout, which manifests as a redirection of resources toward specific job aspects, withdrawal from work associates including care recipients (Hobfoll, 2001; Leiter et al., 1998), and a reduction in the effort and vigilance required for high-quality work (Halbesleben et al., 2008).

We adapted the Nursing Worklife Model to better accommodate care aides working in NHs since no comparable model exists for the setting. Specifically, we use the construct of organizational context to conceptualize the work environment since it encompasses similar factors (such as leadership and staffing adequacy) but is less constrained to a particular staff group or setting (e.g., a broader consideration of interactions rather than specifically nurse–physician collaboration). In our model, a weaker organizational context (or a less effective work environment) and exposure to chronic losses (stressors such as exposure to physical or verbal abuse, rushed work schedules, and limited decision-making) increases the risk of burnout among care aides, in turn.

Relation-centered care views the relationship between the care provider and care recipient as the primary determinant of quality. For NH residents, relationships with staff greatly affect their experience within the facility (Bowers et al., 2001; Coughlan & Ward, 2007; Gruneir et al., 2023; Vaismoradi et al., 2016). We posit that when care aides experience symptoms of burnout, they subsequently redirect their resources, specifically time and emotional resources, and they shift their approach to care from a relation-centered approach to task-oriented care. Relation-centered (or relational care) is personhood-focused and makes explicit the need to recognize the emotional needs of both carers and care receivers and their influence on one another (Beach et al., 2006). Fernandez-Basanta and colleagues refer to this as “caring beyond technique,” which care staff are able to fulfill when their own emotional needs are met and both they and their care recipients are fully humanized (Fernández-Basanta et al., 2023). When care staff are required to suppress their emotions due to time constraints, they experience emotional exhaustion and subsequently protect themselves by disengaging (depersonalizing) and reducing their personal exposure to care recipient suffering. This results in a shift to task-oriented care, which prioritizes efficiency and getting a job done over human connections among care staff and care recipients. The shift from relation-centered to task-oriented care is not a shift in specific care activities but rather in the approach to engaging with care recipients and the provision of care. In our conceptual framework, the symptoms of burnout, most notably emotional exhaustion and depersonalization, result in care aides having less emotional capacity to establish meaningful connections with residents through care activities; instead, care aides approach care activities as tasks to complete.

## Method

### Design and Data

This is a repeated cross-sectional study that uses data from the Translating Research in Elder Care (TREC) research program (www.trecresearch.ca). The goal of the TREC program is to conduct research that contributes to the provision of quality care for NH residents and the quality of work–life for care staff. The TREC program has been collecting data in Western Canadian NHs since 2009. For this study, we focused on data collected over Waves 3 (September 8, 2014–May 15, 2015), Wave 4 (May 1, 2017–December 19, 2017), and Wave 5 (September 1, 2019–March 10, 2020). NHs in five health regions across three provinces (Calgary and Edmonton zones in Alberta, Interior Health and Fraser Health in British Columbia, and the Winnipeg Regional Health Authority in Manitoba) were randomly sampled based on owner-operator model and bed size (Estabrooks et al., 2009; Rycroft-Malone et al., 2009).

Two types of data are held within the TREC program. The first is referred to as the TREC Survey, a suite of surveys designed to measure various aspects of the work environment (at the facility and care unit levels) and the work experience of different types of staff members. For this study, we relied primarily on the care aide survey. The care aide survey was administered using in-person computer-assisted interviews by trained data collectors (Squires et al., 2013). Care aides were eligible to participate in the survey if they: (a) had been employed for at least 3 months in the NH; (b) were assigned to a specific unit at least 50% of the time; and (c) had worked three or more shifts in the prior month. Care aides were surveyed on their demographics, work history, and work–life, including measures such as job satisfaction, mental and physical health, and burnout. Due to the sampling strategy and inclusion criteria, we were able to attach each care aide to the specific care unit in which they worked. TREC Survey data has been frequently used to characterize the NH workforce (Chamberlain et al., 2019; Estabrooks et al., 2015), describe their work–life experiences (Hoben et al., 2017; Squires et al., 2015), and support quality improvement interventions (Cranley et al., 2022; Estabrooks et al., 2016; Wagg et al., 2023).

The second type of data held within TREC used for this study is the routinely collected Resident Assessment Instrument Minimum Data Set 2.0 (RAI-MDS 2.0). The RAI-MDS 2.0 is a clinical assessment mandated for completion on all NH residents within the TREC participating regions at admission, subsequent quarterly intervals, and following major health changes (Hirdes et al., 2011). The RAI-MDS 2.0 contains over 400 items on resident characteristics including diagnoses, physical functioning, cognitive performance, responsive behaviors, and certain treatments. The assessment is completed by care staff as part of usual care processes and is regularly used for research and quality reporting purposes (Doupe et al., 2018; Fries et al., 1997; Mor et al., 2011). The RAI-MDS 2.0 released to TREC can be linked to the TREC Survey at both the facility and unit level.

This research was approved by the Research Ethics Board at the University of Alberta (Pro00037937).

### Burnout

Burnout among care aides was measured using the Maslach Burnout Inventory (MBI)—General Survey (short-form; Leiter & Maslach, 2016; Maslach & Jackson, 1981). The MBI consists of three sub-scales: emotional exhaustion (the central hallmark of burnout), cynicism (negative feelings toward the work, also known as depersonalization), and diminished professional efficacy (feelings about the ability to carry out the work, also known as “personal accomplishment” in other literature). The MBI is a series of Likert-type scale questions on the frequency (never—daily) of specific work-related feelings; sub-scale scores are derived from the mean of items for a 7-point score from zero to six, with higher scores indicating greater burnout. Sub-scales are intended to be reported separately (rather than as a single scale) and are not necessarily correlated, reflecting both the multi-dimensional and varied experience of burnout. The MBI was constructed from multiple data sources including case studies and interviews and since its introduction in the 1980s, it has become one of, if not the, most widely used assessment for burnout (Bianchi et al., 2019). It has been validated for use among various health and education workers and in multiple languages (Byrne, 1994; Pisanti et al., 2013; Shoman et al., 2021). In this study, we report all three sub-scales in the descriptive characteristics but include only emotional exhaustion and cynicism sub-scales in later analyses. We chose this strategy because earlier work (Chamberlain et al., 2017) and our preliminary analyses showed very low scores on diminished professional efficacy, indicating that care aides had confidence in their work-related abilities, and very little variability in our sample, which would challenge the models (Hoben et al., 2023). We characterized high emotional exhaustion as a score >3.00 and high cynicism as a score >2.33, as consistent with prior work (Chamberlain et al., 2017).

We estimated the percentage of care aides in each unit with MBI sub-scale scores above the designated cut-offs for emotional exhaustion (scale scores >3.00) and cynicism (scale scores >2.33). We considered other strategies including estimating the mean scale scores for each unit but found that this strategy was the most consistent with our conceptual approach to using the MBI scales and offered the greatest variation for statistical purposes.

### Resident Outcomes

From a previously generated list of 13 practice-sensitive resident outcomes derived from the RAI-MDS 2.0 (Estabrooks et al., 2013), we identified three outcomes that we believe are manifestations of the negative consequences of care aides redirecting resources away from residents (and consequently shifting their approach to care): antipsychotic use without indication, depressive symptoms, and responsive behaviors. All three outcomes result from clinical situations in which an intimate understanding of the resident’s emotional and physical states requires considerable time to manage. When care aides are overloaded, they lack the resources (time, attention, and emotional capacity) to assess and engage in a relation-centered way. Instead, a task-based approach means less attention to residents’ emotional states and a greater emphasis on medicalized approaches to symptom management (such as antipsychotic prescribing). Further, residents with impaired communication require particularly close attention to their non-verbal cues to identify mood or other needs, which is difficult when care aides have limited time and attention for one-on-one meaningful engagement.

Although care aides are not responsible for antipsychotic prescribing decisions, care aides interact most frequently with residents and these interactions along with care aide reporting of these interactions do influence prescribing. Depressive symptoms are highly prevalent in NHs and have been shown to be strongly influenced by opportunities for meaningful social engagement, which heavily relies on care aide engagement. Finally, responsive behaviors are believed to be the result of unmet needs that the resident cannot otherwise express. As the primary frontline providers, care aides attend to the majority of residents’ needs but also respond to various behaviors reflective of emergent needs. While each outcome has multiple and varied precipitating factors, they are each impacted by the day-to-day care aide practices that shape the resident experience and the quality of their interactions. As per extant theory and research referenced in the prior section, we anticipate that care aides experiencing burnout will be less able to contribute to a socially fulfilling environment and less able to recognize resident cues or respond to them in a timely way. This results in a greater likelihood of behavioral and psychiatric symptoms among residents.

Antipsychotic use without indication was defined as any use of an antipsychotic medication in the seven days prior to assessment without a concurrent diagnosis of psychosis. Depressive symptoms were measured using the RAI-MDS 2.0-embedded Depression Rating Scale (DRS) which includes items on expressed mood, where a score of three or greater suggests the presence of depressive symptoms (Burrows et al., 2000). Responsive behaviors were measured as the presence of any of inappropriate behaviors, verbally or physically abusive behaviors, or resistance to care within the seven days prior to assessment. Outcomes were at the resident level and were not aggregated to the care unit level.

### Analysis

We characterized facilities, units, care aides, and residents using descriptive statistics for each wave of data collection included in the study. Facilities were described by owner-operator model (for-profit, not-for-profit), size (small: <80 beds, medium: 80–120 beds, large: >120 beds), and province. Units were described by type (general, dementia, mental health, or other), unit size, the number of care aides assigned to the unit who completed the TREC Survey, and organizational context as measured by the Alberta Context Tool, which was developed to assess care providers’ perceptions of context as derived from the PARiHS framework that consists of three constructs: culture, leadership, and evaluation (Estabrooks et al., 2009). We also plotted the percentage of care aides per unit with high emotional exhaustion and cynicism. Care aides were described by age at the time of the survey, sex, training and time in the job (tenure), job satisfaction, mental and physical health as measured by the SF-8, the frequency of rushed and incomplete tasks on the prior shift, and exposure to dementia-related responsive behaviors over the five prior shifts. Residents were characterized by their demographics, cognitive performance as measured by the Cognitive Performance Scale (CPS; Morris et al., 1994), physical function as measured by the Activities of Daily Living (ADL) Hierarchy Scale (Morris et al., 1999), select medical diagnoses, physical restraint use, indwelling catheter use, pain, as well as the reported outcome measures.

To estimate associations between care aide burnout and resident outcomes, we constructed six multi-level logistic regression models. We modeled each combination of emotional exhaustion and cynicism (independent variables) and resident outcome (dependent variable) separately (3 outcomes × 2 independent variables = 6 models). Models nested residents (the unit of analysis) within units and units within facilities. Emotional exhaustion and cynicism were entered into the models as the percentage of care aides in a care unit above the specified cut-off. For interpretation, we multiplied the estimated regression effects of emotional exhaustion and cynicism by 10 to obtain the association between a 10 percentage point change of each unit-aggregated burnout score and each of the three resident outcomes, as was done by Cimiotti and colleagues (2012). We first ran an unadjusted model and then added potentially confounding variables in sequential blocks to assess for changes in our primary estimates of interest. The study variables were grouped based on prior theoretical and empirical research: resident demographics, resident health characteristics, care aide demographics including time working as a care aide, care aide work–life, unit characteristics, and facility characteristics. We did not control for unit-level staffing since it did not meet the criteria for confounding.

## Results

Our sample consisted of 290, 309, and 282 units in 88, 93, and 87 facilities for each of Waves 3, 4, and 5, respectively. In all waves, facilities were largely private for-profit ownership (45.4%, 43.0%, 41.4%) and defined as large (120+ beds, 43.2%, 39.8%, 40.2%). The majority of units were designated for general care (69.3%, 66.7%, 69.2%) with a mean of 37.2 (*SD* = 4.8), 36.7 (*SD* = 5.1), and 34.0 (*SD* = 16.0) beds (see Table 1).

**Table 1.** Facility and Unit Descriptive Characteristics Across Waves 3, 4, and 5 From the TREC Survey.

Facility and unit characteristics	Wave 3 (Sept 2014–May 2015)	Wave 4 (May 2017–Dec 2017)	Wave 5 (Sept 2019–Mar 2020)
Facilities	*N* = 88	*N* = 93	*N* = 87
Owner-operator model, *N* (%)			
Public not-for-profit	16 (18.2)	19 (20.4)	20 (23.0)
Private for-profit	40 (45.4)	40 (43.0)	36 (41.4)
Voluntary	32 (36.4)	34 (36.6)	31 (35.6)
Size (beds), *N* (%)			
Small (35–79)	20 (22.7)	20 (21.5)	20 (23.0)
Medium (80–119)	30 (34.1)	36 (38.7)	32 (36.8)
Large (120+)	38 (43.2)	37 (39.8)	35 (40.2)
Location, *N* (%)			
Alberta	33 (37.5)	35 (37.6)	33 (37.9)
British Columbia	39 (44.3)	42 (45.2)	38 (43.7)
Manitoba	16 (18.2)	16 (17.2)	16 (18.4)
Units	*N* = 290	*N* = 309	*N* = 282
Type, *N* (%)			
General	201 (69.3)	206 (66.7)	195 (69.2)
Dementia	61 (21.0)	59 (19.1)	51 (18.1)
Mental health	3 (1.0)	3 (1.0)	3 (1.1)
Other	25 (8.6)	41 (13.3)	33 (11.7)
Size			
*M* (*SD*)	37.2 (4.8)	36.7 (5.1)	34.0 (16.0)
Minimum–maximum	12–101	8–46	12–110
Organizational context (ACT scale scores)^a^, *M* (*SD*)			
Leadership	3.9 (0.2)	4.0 (0.2)	4.0 (0.2)
Culture	4.1 (0.2)	4.1 (0.2)	4.1 (0.2)
Evaluation	3.7 (0.2)	3.8 (0.2)	3.8 (0.2)
Formal interactions	1.4 (0.3)	1.4 (0.3)	1.5 (0.3)
Informal interactions	4.1 (0.6)	4.1 (0.6)	4.1 (0.6)
Social capital	4.0 (0.2)	4.0 (0.2)	4.0 (0.2)
Structural resources	2.7 (0.8)	2.7 (0.8)	2.6 (0.7)
Organizational slack (staff)	2.9 (0.6)	2.9 (0.6)	2.8 (0.6)
Organizational slack (space)	3.5 (0.7)	3.5 (0.7)	3.5 (0.7)
Organizational slack (time)	3.4 (0.4)	3.4 (0.4)	3.5 (0.4)

*Note.* TREC = Translating Research in Elder Care; *SD* = standard deviation.

a ACT = Alberta Context Tool (scale scores derived by taking the mean of Likert-type responses for 2–9 items, depending on scale).

At each wave, the sample included 3,834, 3,985, and 3,547 care aides, respectively. Within each wave, the majority of care aides were 30 to 49 years old (52.9%, 53.3%, 53.4%) and female (89.8%, 89.2%, 89.3%). Care aides rated high levels of job satisfaction, with a mean above 4.0 at each wave, and reported frequent rushed tasks (mean [*SD*] of 2.7 [2.6], 2.8 [2.7], and 2.9 [2.8]) and tasks left undone (1.5 [1.9], 1.6 [1.9], and 1.6 [2.1]) on their last shift (see Table 2).

**Table 2.** Care Aide Descriptive Characteristics Across Waves 3, 4, and 5 From the TREC Survey.

	Wave 3 (Sept 2014–May 2015)	Wave 4 (May 2017–Dec 2017)	Wave 5 (Sept 2019–Mar 2020)
Care aide characteristics	*N* = 3,834	*N* = 3,985	*N* = 3,547
Age (years), *N* (%)			
<30	393 (10.2)	390 (9.8)	296 (8.3)
30–49	2,027 (52.9)	2,123 (53.3)	1895 (53.4)
50+	1,414 (36.9)	1,472 (36.9)	1356 (38.2)
Female, *N* (%)	3,443 (89.8)	3,549 (89.2)	3165 (89.3)
English as first language, *N* (%)	1,480 (38.6)	1,342 (33.7)	1095 (30.9)
Care Aide Certificate, *N* (%)	3,552 (82.7)	3,727 (93.6)	3318 (93.5)
Years worked as a Care Aide, *M* (*SD*)	11.0 (9.0)	11.6 (9.0)	11.9 (9.0)
Years on unit, *M* (*SD*)	5.6 (5.9)	5.8 (5.9)	6.2 (6.3)
Job satisfaction^a^, *M* (*SD*)	4.2 (0.6)	4.2 (0.6)	4.3 (0.6)
Mental health^b^, *M* (*SD*)	52.0 (8.3)	51.9 (8.5)	51.4 (8.7)
Physical health^b^, *M* (*SD*)	49.6 (8.2)	49.1 (8.2)	48.6 (8.1)
Number of rushed tasks on last shift, *M* (*SD*)	2.7 (2.6)	2.8 (2.7)	2.9 (2.8)
Number of undone tasks on last shift, *M* (*SD*)	1.5 (1.9)	1.6 (1.9)	1.6 (2.1)
Number of dementia-related responsive behaviors exposed to over last 5 shifts, *M* (*SD*)	3.2 (1.7)	3.3 (1.6)	3.2 (1.7)
Maslach Burnout Inventory sub-scales, *N* (%)			
High emotional exhaustion (score > 3.00)	1,404 (36.8)	1,540 (38.7)	1,504 (42.5)
High cynicism (score > 2.33)	1,879 (50.0)	1,988 (50.2)	1,796 (50.9)
Low efficacy (score < 3.3)	72 (2.0)	111 (2.3)	86 (2.4)

*Note*. TREC = Translating Research in Elder Care; *SD* = standard deviation.

a Job satisfaction: single-item instrument with a 5-point Likert-type scale response (higher scores correspond to higher job satisfaction). ^b^Eight-Item Short Form Survey (SF-8) mental and physical health summary scales, respectively (scores from 0 [worst health] to 100 [best health]).

**Figure 2.**
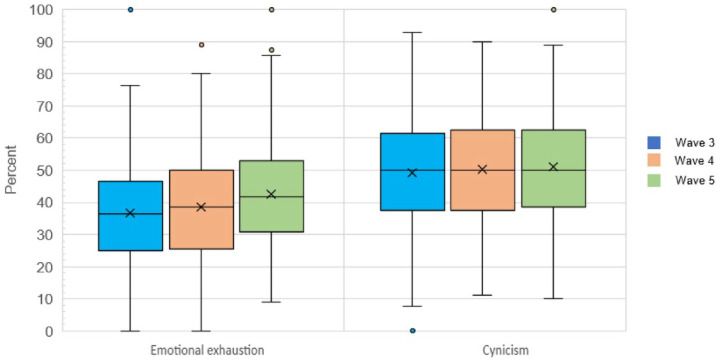
Distribution of Percentage of Care Aides Per Unit that Reported High Emotional Exhaustion and High Cynicism.

In Figure 2, we provide the distribution of the proportion of care aides that experienced emotional exhaustion and cynicism across care units, by wave. The proportion of care aides reporting diminished professional efficacy at each wave was very low (mean [*SD*]: 1.9 [4.4], 2.7 [4.7], and 2.4 [4.6]), data not shown.

There were 10,637, 10,927, and 10,117 residents at each wave. Residents had a mean (*SD*) age of 84.6 (10.2), 84.8 (10.5), and 84.8 (10.6) years and were predominantly female (68.2%, 66.6%, and 66.3%). A minority of residents exhibited no or mild cognitive impairment (18.9%, 18.5%, and 19.9%) or minimal impairment in ADL (9.8%, 7.9%, and 7.5%). Responsive behaviors were common with resists care being the most frequently reported. Of the practice-sensitive outcome measures, 21.1%, 17.8%, and 18.3% of residents had antipsychotic use without indication, 27.9%, 23.6%, and 20.7% exhibited depressive symptoms, and 39.6%, 39.0%, and 38.5% showed any responsive behaviors at each time point, respectively (see Table 3).

**Table 3.** Resident Descriptive Characteristics From the Time Periods Corresponding With Wave 3, Wave 4, and Wave 5 of the TREC Survey.

	Wave 3 (Sept 2014–May 2015)	Wave 4 (May 2017–Dec 2017)	Wave 5 (Sept 2019–Mar 2020)
Resident characteristics	*N* = 10,637	*N* = 10,927	*N* = 10,117
Age, *M* (*SD*)	84.6 (10.2)	84.8 (10.5)	84.8 (10.6)
Female, *N* (%)	7,260 (68.2)	7,278 (66.6)	6,712 (66.3)
Length of stay (days), *N* (%)			
<90	1,286 (12.1)	1,294 (11.8)	1,191 (11.8)
90–364	2,331 (21.9)	2,733 (25.0)	2,348 (23.2)
365+	7,020 (66.0)	6,900 (63.2)	6,580 (65.0)
Cognitive impairment, *N* (%)			
None or mild (CPS < 1)	2,012 (18.9)	2,021 (18.5)	2,010 (19.9)
Moderate (CPS = 2–3)	5,338 (50.2)	5,569 (51.0)	5,116 (50.6)
Severe (CPS = 4–6)	3,286 (30.9)	3,336 (30.5)	2,993 (29.6)
Activities of daily living impairment, *N* (%)			
Minimal	1,046 (9.8)	857 (7.9)	764 (7.5)
Moderate	4,464 (42.0)	4,440 (40.6)	3,700 (36.6)
Dependent	5,126 (48.2)	5,627 (51.5)	5,655 (55.9)
Responsive behaviors, *N* (%)			
Inappropriate behavior	1,654 (15.6)	1,592 (14.6)	1,451 (14.4)
Verbally abusive	1,848 (17.4)	1,759 (16.12)	1,564 (15.4)
Physically abusive	1,219 (11.5)	1,284 (11.8)	1,083 (10.7)
Wandering	1,932 (18.2)	1,739 (15.9)	1,488 (14.7)
Resists care	3,173 (29.9)	3,261 (29.9)	2,985 (29.5)
Selected diagnoses, *N* (%)			
Alzheimer’s disease or other dementia	6,648 (62.5)	6,797 (62.2)	6,230 (61.6)
Chronic obstructive pulmonary disease	1,537 (14.5)	1,543 (14.2)	1,437 (14.3)
Congestive heart failure	1,277 (12.0)	1,543 (11.9)	1,235 (12.3)
Depression	3,094 (29.1)	3,264 (29.9)	3,283 (32.0)
Diabetes mellitus	2,244 (21.1)	2,350 (21.5)	2,252 (22.3)
Renal failure	725 (6.8)	855 (7.9)	843 (8.4)
Stroke	2,099 (19.7)	2,206 (20.2)	1,973 (19.5)
Practice-sensitive outcome measures			
Antipsychotic use without indication, *N* (%)	2,247 (21.1)	1,943 (17.8)	1,852 (18.3)
Depressive symptoms (DRS > 3)	2,964 (27.9)	2,575 (23.6)	2,089 (20.7)
Presence of responsive behaviors^a^	4,207 (39.6)	4,260 (39.0)	3,891 (38.5)

*Note. SD* = standard deviation; CPS = Cognitive Performance Scale; DRS = Depression Rating Scale.

a Does not include wandering.

From the multi-level logistic regression models, none of the variables showed any associations in any of the six unadjusted models and this was consistent following adjustment for resident (age, sex, cognitive impairment, ADL impairment, dementia), care aide (age, sex, years worked on unit, mental health, physical health, care tasked rushed on last shift, care tasks undone on last shift), unit (type, size), and facility (owner-operator model, size) characteristics. Model results, all of which indicate no association, are shown in Table 4.

**Table 4.** Estimates of Association Between Percentage of Care Aides Working on Unit Experiencing Burnout (Emotional Exhaustion or Cynicism) and Practice-Sensitive Outcomes Among Residents Living on the Same Unit, Unadjusted and Adjusted Estimates Shown.

	Antipsychotics without indication Odds ratio (95% Confidence interval)		Depressive symptoms Odds ratio (95% Confidence interval)		Responsive behaviors Odds ratio (95% Confidence interval)	
Burnout measures	Unadjusted	Adjusted^a^	Unadjusted	Adjusted^a^	Unadjusted	Adjusted^a^
Emotional exhaustion	0.99 [0.97, 1.02]	1.01 [0.98, 1.04]	0.99 [0.97, 1.02]	0.97 [0.94, 1.00]	0.99 [0.96, 1.01]	1.00 [0.97, 1.02]
Cynicism	0.99 [0.96, 1.01]	1.00 [0.97, 1.02]	0.99 [0.97, 1.01]	0.98 [0.95, 1.00]	1.00 [0.98, 1.02]	1.02 [0.99, 1.04]

a Adjusted for resident characteristics—age, sex, cognitive impairment, activities of daily living impairment, dementia diagnosis; care aide characteristics—age, sex, years worked on the unit, mental health, physical health, care tasks rushed on last shift, care tasks undone at last shift; unit characteristics—type, size; facility characteristics—owner-operator model, size.

## Discussion

Using a large sample of care aides working in NHs, we found that over one-third reported emotional exhaustion and one-half reported high cynicism but that very few reported diminished professional efficacy. Among our sample of residents, approximately 20% had received an antipsychotic without indication, 20% experienced depressive symptoms, and 40% of residents had exhibited responsive behaviors. Our analysis showed no association between unit-level burnout among care aides and the practice-sensitive outcomes among residents that we included in our study.

The Conservation of Resources model posits that the depletion of personal resources due to work-related stressors leads care workers to concentrate their efforts on specific tasks while limiting or eliminating effort on more emotionally demanding tasks, consequently resulting in reduced quality of care (Hobfoll, 2001). We used routinely measured and widely reported outcomes, which may represent care tasks where care aides continue to invest effort even as their resources dwindle. It is possible that outcomes that are not included in routine reporting, for example, quality of life, would have been more sensitive to differences in care aide behavior.

At the same time, however, our outcomes (depressive symptoms, responsive behaviors, and unindicated antipsychotic use) tap into various aspects of social care and support, areas that are typically deprioritized in favor of physical care activities in the resource-constrained work environments of the NH sector (Ludlow et al., 2021; Renner et al., 2022). One time-motion study found that care aides spent over half of their shift undertaking personal care tasks, typically in 1- to 3-minute segments with frequent interruptions, and nearly one-quarter of their shift entailed work that did not involve residents (Mallidou et al., 2013). Upward of 50% of care aides report missing at least one care task and 60% report rushing at least one care task per shift (Knopp-Sihota et al., 2015; Song et al., 2020). It is possible, then, that given the time constraints under which they generally work, care aides are perpetually unable to address sufficiently residents’ social care and support needs or more broadly engage in relation-centered care approaches to care—regardless of their burnout—and that is why we found no differences in resident outcomes across burnout levels. Future research that incorporates measures of relation-centered care may be better able to tease out the effects of burnout on care outcomes.

We also found that despite the high frequency of emotional exhaustion and cynicism, care aides reported very low levels of diminished professional efficacy. Other researchers have shown that while various personal and organizational factors increase the risk of emotional exhaustion and cynicism, professional efficacy is more aligned with factors that protect against burnout, including self-efficacy (Shoji et al., 2016). Self-efficacy is an individual’s belief about their abilities to manage their work and it is a key personal resource. Professional efficacy and self-efficacy have consistently been found to be highly correlated across diverse study settings, suggesting that high professional efficacy can buffer the effects of heightened emotional exhaustion and cynicism (Leiter, 1992).

We used an adapted version of the Nursing Worklife Model to situate care aides within their work environment. Prior research has shown that various elements of organizational context (work environment) increase the likelihood of emotional exhaustion and cynicism among care aides (Chamberlain et al., 2017). We found that both emotional exhaustion and cynicism were frequently reported by our sample and that their prevalence varied across care units. Although we did not see any changes in the null association between burnout and outcomes following adjustment for work environment variables, future research should explore staff-to-resident outcome relationships in different work environments, especially those differentiated by leadership and care approach.

Research to date is clear that the NH work environment is associated with burnout among staff, which, in turn, leads to missed care (White et al., 2019), reduced tolerance for residents (Àstrom et al., 1990; Neuberg et al., 2017), and greater staff-reported mistreatment of residents (Bužgová & Ivanová, 2011). Others have reported that burnout acts as a mediator (or partial mediator) between nurse staffing and outcomes (Johnson et al., 2017; Laschinger & Leiter, 2006). The research on direct effects is lacking, with most authors extrapolating from findings on the effects of the work environment or staffing on burnout and resident outcomes without empirically establishing a direct link between the two (Aiken et al., 2009; Kutney-Lee et al., 2016). Future research should explore how care aides and nurses experiencing burnout differ in how they conduct their work, especially how they approach care, relative to those without burnout, and the impact on staff and resident outcomes at very high levels of emotional exhaustion and cynicism where high levels of professional or self-efficacy may not pertain.

## Limitations

There are a number of limitations to this research. First, we used burnout scores aggregated to the unit level rather than individual scores since we could not determine specific care relationships. We, therefore, may not have truly captured care aides and the residents under their care. As described above, we may not have selected outcomes that were sufficiently sensitive to the changes in care aides’ work due to burnout; however, it is unclear what outcomes would be better suited given that even measures on patient satisfaction or perceptions of quality have also shown inconsistent results. Third, our sample included only homes in Western Canada, which may mean limited generalizability to other regions; however, our resident and care aide samples are generally consistent with those reported elsewhere. Finally, it is worth noting that approximately 35% of residents appeared in two consecutive waves.

## Conclusion

Burnout among care aides working in NHs was not associated with increased antipsychotic use, depressive symptoms, or responsive behaviors among the residents within their care. While care aides frequently reported emotional exhaustion and cynicism, they also infrequently reported diminished personal accomplishment, which may act as a buffer against burnout. It is still unclear, though, how care aides experiencing burnout carry out their jobs compared to those without burnout and whether this would have been better captured with different resident outcomes. Despite our null findings, understanding the impact of the quality of work–life on care aides and residents is critical to identifying strategies to improve NH care and experience.

## Supplemental Material

sj-docx-1-mcr-10.1177_10775587231220072 – Supplemental material for Burnout Among Nursing Home Care Aides and the Effects on Resident Outcomes Supplemental material, sj-docx-1-mcr-10.1177_10775587231220072 for Burnout Among Nursing Home Care Aides and the Effects on Resident Outcomes by Andrea Gruneir, Stephanie A. Chamberlain, Charlotte Jensen, Greta Cummings, Matthias Hoben, Sheila Boamah, Clarisse Bosco, Sadaf Ekhlas, Sascha R. Bolt, Tim Rappon, Whitney B. Berta, Janet Squires and Carole A. Estabrooks in Medical Care Research and Review
