# Supplementary material for: Burnout Among Nursing Home Care Aides and the Effects on Resident Outcomes
Source: Med Care Res Rev. 2023 Dec 30;81(3):233–44. doi: 10.1177/10775587231220072 (PMC11092296; doi:10.1177/10775587231220072)
Supplement: sj-docx-1-mcr-10.1177_10775587231220072 – Supplemental material for Burnout Among Nursing Home Care Aides and the Effects on Resident Outcomes [file sj-docx-1-mcr-10.1177_10775587231220072.docx]

Appendix Figure 1: Summary of manuscript inclusion and exclusion process

Included

Eligibility

Screening

Identification

Full-text articles assessed for eligibility

n=1189

Articles excluded because of a lack of relevant discussion of burnout and patient outcomes/patient population

n= 1126

Duplicates removed

n=4

Articles excluded because of a lack of relevant discussion of burnout and patient outcomes/patient population in the title, abstract, and key words

n= 7373

Articles with titles, abstracts and keywords screened:

n=8562

Articles identified through search on Ovid MEDLINE, EMBASE; EBSCOhost, CINAHL, and Scopus in 2017 (<2017). An updated search was conducted in February 2020 to include new publications (2018-2020).

n= 8562

Final literature sample

n=59

Duplicates removed as well as non-relevant articles

n= 8

Adjusted

d

Adjusted final sample

n=51

Appendix Figure 2 – Summary of additional review of manuscripts for relevance

Overview

Relevant articles were read, discussed, and considered carefully for type of relevance.

n= 51

Dissertations

Dissertations were grouped by themselves

n= 7

Methods

Articles concerned with methods yet relevant was grouped alone

n=1

Intermediate

Articles that considered Burnout as an Intermediate Variable were grouped together

n=5

Outcome Specific

dOutc

Articles concerned with outcomes were grouped by outcome type

n=38

Job Satisfaction as Outcome

n=1

Provider-Patient Interaction as Outcome

n=6

Burnout as Outcome

n=13

Staff reported Resident Outcomes

n=14

Clinical Outcomes

n=3

Satisfaction as Outcome

n=1

Appendix Table 1 – Summary of studies testing for associations between care provider burnout and resident/patient outcomes as reported by resident/patient

| **Author** | **Title** | | **Year** | **Country** | **Care Setting** | **Care Provider Type(s)** | **Measures** | **Outcome** | **Findings** |
| --- | --- | --- | --- | --- | --- | --- | --- | --- | --- |
| Leiter,  M. P. et  al. | The correspondence of patient satisfaction  and nurse burnout | | 1998 | Canada | Hospital | Nurses | MBI-General  Survey - subscale:  emotional  exhaustion,  cynicism, professional efficacy | Patient satisfaction  (Patient Judgments of Hospital Quality Questionnaire) | - Nurse exhaustion correlated with lower patient satisfaction with nurses and other aspects of care - Nurse cynicism associated with lower patient satisfaction with nurses - No correlations for professional efficacy and outcomes |
| Chao, M.;  Shih, C. T.;  Hsu, S. F. | Nurse occupational burnout and patient-rated quality of care: The boundary conditions of emotional intelligence  And demographic profiles | | 2016 | Taiwan | Hospital | Nurses | MBI – Chinese version (subscales not specified) | Quality of care assessment by:   - Patients - Family members of patients - Employed caregivers | - Nurses in contract positions (vs. permanent) and nurses with less experience were perceived to provide lower quality of care when experiencing burnout |
| Cimiotti,  J.P. et al. | Nurse staffing,  burnout, and health care -associated infection. | 2012 | | USA | Hospital | Registered nurses | MBI-Human Health Services – subscale:  emotional exhaustion | Infections:   - Catheter-associated urinary tract - Surgical site | - 10% increase in the proportion of nurses in a hospital experiencing emotional exhaustion associated with greater infections |
| Chao, S-F | Does geriatric nursing staff burnout predict well-being of LTC residents? | 2019 | | Taiwan | Long-term care facilities | Geriatric nursing staff | MBI-Human Health Services – subscale:  emotional exhaustion,  de-personalization,  professional accomplishment | Resident well-being:   - Satisfaction - Perceived quality of life - Depressive symptoms | - Higher depersonalization among nurses was associated with worse resident well-being across all measures - No associations with either emotional exhaustion or personal accomplishment |

MBI=Maslach Burnout Inventory

Appendix Table 2a: Full Model Results – Emotional Exhaustion

|  | Antipsychotics without Indication |  | Depressive Symptoms |  | Responsive Behaviours |  |
| --- | --- | --- | --- | --- | --- | --- |
|  | Estimate | Odds Ratio  (95% CI) | Estimate | Odds Ratio  (95% CI) | Estimate | Odds Ratio  (95% CI) |
| Intercept | -0.86 | 0.42 (0.1-1.71) | 0.41 | 1.50 (0.33-6.94) | -0.82 | 0.44 (0.12-1.63) |
| Emotional Exhaustion among Care Aides | 0.01 | 1.01 (0.98-1.04) | -0.03 | 0.97 (0.94-1.00) | 0 | 1.00 (0.97-1.02) |
| Resident age | -0.02 | 0.98 (0.97-0.98) | -0.01 | 0.99 | 0 | 1.00 (0.99-1.00) |
| Resident Sex  (reference: male) |  |  |  |  |  |  |
| Female | -0.19 | 0.82 (0.77-0.88) | 0.46 | 1.58 (1.48-1.69) | -0.21 | 0.81 (0.77-0.86) |
| Cognitive Performance Scale | 0.17 | 1.18 (1.15-1.21) | 0.02 | 1.02 (0.99-1.04) | 0.32 | 1.38 (1.35-1.41) |
| ADL Hierarchy Scale | -0.13 | 0.88 (0.86-0.9) | -0.04 | 0.96 (0.94-0.99) | -0.07 | 0.93 (0.91-0.95) |
| Alzheimer’s disease or other dementia | 0.60 | 1.82 (1.69-1.96) | 0.13 | 1.14 (1.06-1.22) | 0.41 | 1.51 (1.42-1.61) |
| Care Aide age | 0.01 | 1.01 (1.00-1.02) | -0.02 | 0.98 (0.97-1.00) | -0.02 | 0.98 (0.97-0.99) |
| Proportion of care aides who were female on unit | 0 | 1.00 (0.99-1.02) | 0.01 | 1.01 (1.00-1.01) | 0 | 1.00 (0.98-1.00-1.01) |
| Years worked on unit | -0.02 | 0.98 (0.96-1.00) | -0.02 | 0.98 (0.96-1.01) | 0.02 | 1.02 (1.00-1.04) |
| Mental health | 0 | 1.00 (0.99-1.02) | 0 | 1.00 (0.99-1.02) | 0.01 | 1.01 (0.99-1.02) |
| Physical health | 0.01 | 1.01 (1.00-1.03) | -0.02 | 0.98 (0.96-1.00) | 0 | 1.00 (0.98-1.01) |
| Rushed care tasks | 0.02 | 1.02 (0.97-1.08) | 0.04 | 1.04 (0.99-1.09) | 0 | 1.00 (0.96-1.05) |
| Care tasks left undone | -0.03 | 0.97 (0.91-1.04) | 0.07 | 1.07 (0.99-1.15) | 0 | 1.00 (0.94-1.06) |
| Unit type  (reference: general unit) |  |  |  |  |  |  |
| Non-Secure Dementia | 0.03 | 1.03 (0.81-1.31) | -0.29 | 0.75 (0.58-0.97) | 0.03 | 1.03 (0.82-1.28) |
| Secure Dementia | 0.59 | 1.80 (1.57-2.06) | 0.40 | 1.49 (1.25-1.79) | 0.73 | 2.07 (1.78-2.40) |
| Psychiatric | 0.65 | 1.92 (1.16-3.17) | 0.84 | 2.31 (1.05-5.1) | 0.57 | 1.77 (0.93-3.36) |
| Other | 0.34 | 1.40 (1.20-1.64) | -0.34 | 0.71 (0.60-0.84) | 0.13 | 1.14 (0.98-1.32) |
| Number of beds on unit | -0.01 | 0.99 (0.99-1.00) | -0.01 | 0.99 (0.99-1.00) | 0 | 1.00 (0.99-1.00) |
| Owner-operator model  (reference: public, not for profit) |  |  |  |  |  |  |
| Private for-profit | 0 | 1.00 (0.76-1.31) | -0.64 | 0.53 (0.34-0.83) | -0.26 | 0.77 (0.57-1.03) |
| Not for profit | -0.07 | 0.93 (0.72-1.21) | -0.40 | 0.67 (0.45-1.00) | -0.02 | 0.98 (0.74-1.29) |
| Facility Size  (reference: small) |  |  |  |  |  |  |
| Large | -0.13 | 0.88 (0.67-1.16) | -0.08 | 0.93 (0.57-1.50) | 0.10 | 1.10 (0.81-1.50) |
| Medium | -0.05 | 0.96 (0.72-1.26) | -0.15 | 0.86 (0.53-1.40) | 0.19 | 1.20 (0.88-1.65) |
|  |  |  |  |  |  |  |

CI=confidence interval; ADL=activities of daily living

Appendix Table 2b: Full Model Results – Cynicism

|  | Antipsychotics without Indication |  | Depressive Symptoms |  | Responsive Behaviours |  |
| --- | --- | --- | --- | --- | --- | --- |
|  | Estimate | Odds Ratio  (95% CI) | Estimate | Odds Ratio  (95% CI) | Estimate | Odds Ratio  (95% CI) |
| Intercept | -0.63 | 0.53 (0.14-1.99) | 0.96 | 2.62 (0.61-11.25) | -1.06 | 0.35 |
| Cynicism among Care Aides | 0 | 1.00 (0.97-1.02) | -0.02 | 0.98 (0.95-1.00) | 0.02 | 1.02 (0.99-1.04) |
| Resident age | -0.02 | 0.98 (0.97-0.98) | -0.01 | 0.99 | 0 | 1.00 (0.99-1.00) |
| Resident Sex  (reference: male) |  |  |  |  |  |  |
| Female | -0.19 | 0.82 (0.77-0.88) | 0.46 | 1.58 (1.48-1.69) | -0.21 | 0.81 (0.77-0.86) |
| Cognitive Performance Scale | 0.17 | 1.18 (1.15-1.21) | 0.02 | 1.02 (0.99-1.04) | 0.32 | 1.38 (1.35-1.41) |
| ADL Hierarchy Scale | -0.13 | 0.88 (0.86-0.90) | -0.04 | 0.96 (0.94-0.99) | -0.07 | 0.93 (0.91-0.95) |
| Alzheimer’s disease or other dementia | 0.60 | 1.82 (1.69-1.96) | 0.13 | 1.14 (1.07-1.23) | 0.41 | 1.51 (1.42-1.61) |
| Care Aide age | 0.01 | 1.01 (1.00-1.02) | -0.02 | 0.98 (0.96-1.01) | -0.02 | 0.98 (0.97-0.99) |
| Proportion of care aides who were female on unit | 0 | 1.00 (1.00-1.01) | 0.01 | 1.01 (1.00-1.01) | 0 | 1.00 (1.00-1.01) |
| Years worked on unit | -0.02 | 0.98 (0.96-1.00) | -0.02 | 0.98 (0.96-1.01) | 0.02 | 1.02 (1.00-1.04) |
| Mental health | 0 | 1.00 (0.98-1.02) | 0 | 1.00 (0.99-1.02) | 0.01 | 1.01 (1.00-1.03) |
| Physical health | 0.01 | 1.01 (0.99-1.03) | -0.02 | 0.98 (0.96-0.99) | 0 | 1.00 (0.98-1.01) |
| Rushed care tasks | 0.03 | 1.03 (0.98-1.08) | 0.03 | 1.03 (0.97-1.08) | 0 | 1.00 (0.98-1.01) |
| Care tasks left undone | -0.03 | 0.97 (0.91-1.04) | 0.06 | 1.06 (0.99-1.14) | -0.01 | 0.99 (0.93-1.06) |
| Unit type  (reference: general unit) |  |  |  |  |  |  |
| Non-Secure Dementia | 0.03 | 1.03 (0.81-1.31) | -0.30 | 0.74 (0.57-0.96) | 0.02 | 1.02 (0.82-1.28) |
| Secure Dementia | 0.58 | 1.79 (1.57-2.05) | 0.40 | 1.49 (1.25-1.78) | 0.74 | 2.09 (1.80-2.42) |
| Psychiatric | 0.63 | 1.88 (1.14-3.11) | 0.80 | 2.22 (1.02-4.87) | 0.59 | 1.80 (0.95-3.42) |
| Other | 0.34 | 1.40 (1.20-1.63) | -0.34 | 0.71 (0.61-0.84) | 0.14 | 1.15 (0.99-1.33) |
| Number of beds on unit | -0.01 | 0.99 (0.99-1.00) | -0.01 | 0.99 (0.98-1.00) | 0 | 1.00 (0.99-1.00) |
| Owner-operator model  (reference: public, not for profit) |  |  |  |  |  |  |
| Private for-profit | 0.01 | 1.01 (0.77-1.32) | -0.66 | 0.52 (0.33-0.81) | -0.28 | 0.76 (0.57-1.02) |
| Not for profit | -0.07 | 0.93 (0.72-1.21) | -0.41 | 0.67 (0.45-0.99) | -0.03 | 0.97 (0.74-1.28) |
| Facility Size  (reference: small) |  |  |  |  |  |  |
| Large | -0.12 | 0.88 (0.67-1.16) | -0.08 | 0.92 (0.57-1.49) | 0.09 | 1.10 (0.81-1.49) |
| Medium | -0.04 | 0.96 (0.72-1.27 | -0.15 | 0.86 (0.53-1.40) | 0.18 | 1.20 (0.87-1.64) |
|  |  |  |  |  |  |  |

CI=confidence interval; ADL=activities of daily living
